# Patterns of Volatile Diversity Yield Insights Into the Genetics and Biochemistry of the Date Palm Fruit Volatilome

**DOI:** 10.3389/fpls.2022.853651

**Published:** 2022-03-14

**Authors:** Jonathan M. Flowers, Khaled M. Hazzouri, Alain Lemansour, Tiago Capote, Muriel Gros-Balthazard, Sylvie Ferrand, Marc Lebrun, Khaled M. A. Amiri, Michael D. Purugganan

**Affiliations:** ^1^Center for Genomics and Systems Biology, New York University Abu Dhabi, Abu Dhabi, United Arab Emirates; ^2^Khalifa Center for Genetic Engineering and Biotechnology, United Arab Emirates University, Al Ain, United Arab Emirates; ^3^Date Palm Research and Development Unit, UAE University, Al Ain, United Arab Emirates; ^4^CIRAD, UMR Qualisud, Montpellier, France; ^5^Qualisud, Univ Montpellier, Avignon Université, CIRAD, Institut Agro, IRD, Université de La Réunion, Montpellier, France; ^6^Department of Biology, College of Science, UAE University, Al Ain, United Arab Emirates; ^7^Center for Genomics and Systems Biology, New York University, New York, NY, United States

**Keywords:** volatile organic compound, aroma, flavor, fatty acid ester, metabolic network, volatilome

## Abstract

Volatile organic compounds are key components of the fruit metabolome that contribute to traits such as aroma and taste. Here we report on the diversity of 90 flavor-related fruit traits in date palms (*Phoenix dactylifera* L.) including 80 volatile organic compounds, which collectively represent the fruit volatilome, as well as 6 organic acids, and 4 sugars in tree-ripened fruits. We characterize these traits in 148 date palms representing 135 varieties using headspace solid-phase microextraction gas chromatography. We discovered new volatile compounds unknown in date palm including 2-methoxy-4-vinylphenol, an attractant of the red palm weevil (*Rhynchophorus ferrugineus* Olivier), a key pest that threatens the date palm crop. Associations between volatile composition and sugar and moisture content suggest that differences among fruits in these traits may be characterized by system-wide differences in fruit metabolism. Correlations between volatiles indicate medium chain and long chain fatty acid ester volatiles are regulated independently, possibly reflecting differences in the biochemistry of fatty acid precursors. Finally, we took advantage of date palm clones in our analysis to estimate broad-sense heritabilities of volatiles and demonstrate that at least some of volatile diversity has a genetic basis.

## Introduction

Fruit quality traits have been subject to selection by farmers and breeders since the origin of fruit crop agriculture. This process has contributed to the evolution of physical characteristics of fruits such as their size (Fuller, 2018) and to changes in chemosensory traits including fruit flavor compounds that distinguish domesticated species from their crop wild relatives (Aharoni et al., 2004). Flavor-related traits in fruit crops are an important component of fruit quality (Song and Forney, 2008) that include organic acids and sugars that influence taste and volatile organic compounds (VOCs) that determine fruit aroma. In some cases, selection on flavor traits has improved the palatability of fruits (Tadmor et al., 2002; Aharoni et al., 2004), while in others, intense breeding has contributed to a loss of flavor (Klee and Tieman, 2018).

Date palms are a subtropical fruit crop that consists of approximately 3,000 vegetatively propagated cultivars (Zaid and Arias-Jiménez, 1999), or varieties, that include a small number of commercially important elite cultivars that are valued primarily for their sweet, nutritious fruit (Ghnimi et al., 2017). The domesticated crop is divided broadly into two gene pools consisting of a Western population in North Africa and an Eastern population in West Asia (Arabnezhad et al., 2012; Hazzouri et al., 2015), each defined by distinct genetic ancestries (Flowers et al., 2019). The fruits (“dates”) are a drupe with a lignified stone surrounded by a sugar-rich fibrous pulp whose commercial grade depends on their appearance, texture, and sugar content. Fruits are produced by pollination of female palms in the spring followed by harvest in the summer or fall at either the *khalal* (or *besser*) stage when fruits are unripe and moisture content is at its peak or after the onset of ripening at the *rutab* (ripened) or *tamar* (mature) stage. The *khalal* stage is most familiar to consumers in date growing countries where small numbers of varieties (e.g., “Barhee,” “Um Dhin,” “Khenezi,” and “Hayany”) are consumed at this stage, while dried mature fruits are familiar to consumers worldwide. Fruit development in date palms is generally thought to be climacteric (i.e., ethylene dependent; Abbas and Ibrahim, 1996; Serrano et al., 2001), however, there is evidence that fruits of at least one variety (“Barhee”) may develop in an ethylene-independent fashion (Marondedze et al., 2014).

In date palms, flavor is determined by a complex array of primary and secondary metabolites that vary by fruit developmental stage (Ahmed et al., 1995; Myhara et al., 1999; El Arem et al., 2011; Hatem et al., 2018) and frequently differ quantitatively among varieties (Al-Farsi and Lee, 2008; El Arem et al., 2011). Variation in flavor compounds contributes to diverse flavor profiles (Zaid and de Wet, 2002); for example, some fruits are characterized by nutty flavor notes (e.g., “Asharasi” and “Kenta” varieties; Popenoe, 1913) while others have sweet, honey-like characteristics (e.g., “Halawi”; Popenoe, 1913).

Variation in organic acids is an important determinant of differences in flavor among fruits of date palms. Many studies have reported differences in organic acids among varieties (Al-Farsi et al., 2005; Elshibli and Korpelainen, 2009; Farag et al., 2014; Hamad et al., 2015; Abdul-Hamid et al., 2018; Ghnimi et al., 2018; Kamal-Eldin and Ghnimi, 2018; Hazzouri et al., 2019), and malic acid has been identified as a key acid that differs among date palm fruits and may contribute to flavor differences (Farag et al., 2014; Kamal-Eldin and Ghnimi, 2018).

Sugar composition also varies prominently among varieties (Cook and Furr, 1953; Kanner et al., 1978; Samarawira, 1983; Fayadh and Al-Showiman, 1990; Ahmed et al., 1995; Al-Hooti et al., 1997; Chaira et al., 2009; Elshibli and Korpelainen, 2009; Haider et al., 2014; Hazzouri et al., 2019). Varieties can be classified as “reducing-type” (e.g., “Khalas”), which fully invert sucrose to glucose and fructose during ripening, or “sucrose-type” (e.g., “Sukary”), which deposit sucrose in the mesocarp (i.e., pulp) of the ripened fruit (Dowson and Aten, 1962). While sugar is a strong elicitor of the perception of sweet flavors in date palms, aroma compounds may contribute to the sweet taste of many fruits through the phenomenon of odor-induced changes in taste perception (Djordjevic et al., 2004). In apples, for example, volatiles explain 33% of perceived sweetness suggesting an important role for aroma compounds in either enhancing (“sweet congruent” compounds) or negating sweet perception (Aprea et al., 2017).

Date aroma is determined by a complex mixture of volatiles (Reynes et al., 1996), now referred to as the volatilome, that vary during the course of fruit development (El Arem et al., 2011). Classes of compounds including alcohols, aldehydes, ketones and esters are the most dominant classes of volatile in date palm fruits (El Arem et al., 2011, 2012; Hatem et al., 2018), while aliphatic hydrocarbons may be less important (Jaddou et al., 1984). Many of the most dominant volatiles are derived from fatty-acid and phenylpropanoid pathways and are likely important determinants of date aroma (Khalil et al., 2017). Varieties frequently differ in their volatile composition (Harrak et al., 2005; Khalil et al., 2017; Mezroua et al., 2017; Arif and Lombarkia, 2018; Hatem et al., 2018), but the causes are unknown.

Here we address questions about volatile diversity and biosynthesis that may contribute to differences in date palm flavor-related traits. We profile volatiles in tree-ripened fruit harvested from a panel of 148 palms representing 135 varieties. Our results on volatilome diversity provide new insight into the metabolism of volatiles and other flavor-related compounds in date fruits.

## Materials and Methods

### Fruit Collection

The sampling population used in this study includes both elite and minor cultivars of date palm with most originating from the West Asian population and a smaller number representing the North African gene pool (Hazzouri et al., 2019). The original sampling of tree-ripened fruits from 148 date palms in this study was first reported in Hazzouri et al. (2019; Supplementary Table 1) and data for sugar, organic acid, and moisture content provided in that study. Date palms in this study consisted of adult female fruit-bearing palms located at two farms in the United Arab Emirates. The minimum age range is from roughly 10 years on the Al Falassi farm (Al Schweib, Abu Dhabi, UAE) and 17 years on the farm in Al Hamria (Ras-Al-Khaima, UAE). Both use drip irrigation with ∼750 liters of water per palm every two weeks. The Al Hamria farm uses organic fertilizer (75 kg cow/sheep/chicken dung mix) applied once a year per palm, while the Al Falassi farm is fertilized twice a year with the same mixture. Furthermore, the Al Falassi farm adds potassium sulfate fertilizer at 0.75 kg per palm per year. Finally, the Al Falassi farm is treated with several pesticides (including Dimethoate, Chloropyrifos, Triclorophon, Primiphos Methyl, Malathion) as needed, while there is no phytosanitary treatment in the Al Hamria farm. In both farms, females were pollinated with pollen mixtures from various males that included Ghanami, Sekka, Alkhour, as well as those of unknown provenance.

Fruits of 148 palms used for volatile profiling in this study and for organic acid and sugar quantification in Hazzouri et al. (2019) were allowed to ripen completely to the mature “tamar” stage on the fruit stalk prior to harvest. Ripe fruit in the “tamar” stage was determined by visual inspection and as the stage where fruit abscission occurs. Fruits for different varieties were harvested asynchronously at the abscission stage to allow different varieties to reach the desired developmental stage. Note that for all trees fruit thinning was practiced and the developing fruit stalk bunch covered as standard agronomic practice. Approximately 20–25 fruit were harvested per date palm by removing each fruit from its spike and freezing immediately on dry ice. Fruits were maintained at –40°C up to 24 days prior to shipment on dry ice to Montpellier, France where samples were stored at –20°C prior to volatile quantification.

### Volatile Profiling

Volatile profiling was conducted by repeating the following procedure three times per sample and the outputs treated as technical replicates in downstream analysis. Approximately 10–15 frozen fruits from each of the 148 date palms were crushed and stones removed prior to grinding the fruit flesh to powder on liquid nitrogen. Samples were then stored at –20°C until processing at which time they were divided in two. One part was retained for organic acid and sugar profiling reported previously (Hazzouri et al., 2019) and the remainder for volatile analysis. Volatile compounds were extracted using headspace solid-phase microextraction gas chromatography (SPME/GC). Each sample was analyzed in triplicate. Samples (1 g) were placed in headspace vials (10 mL) with butanol (Sigma-Aldrich) (5 μL/100 mL) as internal standard for semi-quantification.

Extraction was performed at 60°C with 15 min incubation and 60 min trapping on polydimethylsiloxane (PDMS)/DVB (divinylbenzene)/Carboxen fiber (Stableflex™ 50/30μm, Supelco, Bellefonte, Pennsylvania, United States). Analysis was carried out with a gas chromatograph 6890/MSD 5973 system (Agilent Technologies, Palo Alto, United States) with a Gerstel Multipurpose Sampler MPS-2, equipped with a DB-WAX Ultra Inert (UI) polar column 30 m, 0.25 mm, 0.25 μm film thickness (Agilent J&W GC column). Hydrogen was used as carrier gas at 1.2 mL/min at constant flow. Fiber was desorbed at 250°C with the injector port in the gas chromatograph set to Splitless mode. The oven temperature increased from 40°C by 3°C/min to 170°C and then 10°C/min to 240°C. The oven was held at the final temperature for 10 min. The mass spectrometer operated in electron impact (EI+) ionization mode at 70 eV with a scan range of 40–350 Dalton.

Data were analyzed with MassHunter version B 08.00 (Agilent Technologies, Palo Alto, United States). Peaks were identified by comparing the obtained mass spectra with those of the National Institute of Standards and Technology (NIST, Gaithersburg, Maryland, United States) 14 database. Co-injection of series of alkanes, from C8 to C20 (Sigma-Aldrich), was used for the calculation of Kovats retention indices that were then compared with those found in the literature (Pubchem website^1^ and NIST). After careful revision and removal of artifacts and pollution coming from fiber and column, the list of volatiles was reduced to 80 molecules. The raw data consisted of 3 technical replicates of each of the 148 date palm fruit samples and 80 volatiles (see section “Data Availability”).

To allow for sample comparison, semi-quantitative determinations were carried out and expressed in butanol equivalents. The volatile quantification was performed as follows for each technical replicate: m_i_ (μg/100 g dried dates) = K_i_/K_EI_ × A_i_/A_EI_ × m_EI_/m_p_ × 100, where K_i_ is the coefficient of response of the unknown molecule, K_EI_ is the coefficient of response of the internal standard, A_i_ is the peak area of the volatile compound, A_EI_ is the peak area of butanol, m_EI_ is the quantity of butanol, m_p_ is the quantity of date fruit powder. Calculations were performed with setting K_i_/K_EI_ = 1. Our results are therefore a semi-quantification where the concentration is used as a mean of relative comparison pertinent to our study. This semi-quantification allows direct comparison of measurements of each volatile, but precludes rigorous quantification of quantities such as total volatile emissions or volatile emissions per class (e.g., alcohols).

The raw data matrix with three technical replicates was then processed to remove low quality data points using the following procedure. First, any volatile from a single fruit sample with coefficient of variation (CV) > 50% across the three technical replicates was evaluated to determine if one of the technical replicates deviated substantially (as defined below) from the other two. Deviations were calculated for each of the minimum and maximum technical replicates as a percentage of the median replicate as | (replicate—median replicate)| /median replicate. In cases where the minimum or maximum replicates deviated from the median replicate by < 35% and the other by > 55%, the replicate with the larger deviation from the median was excluded. Second, in cases where the CV was > 50% for the three technical replicates, but one of the replicates could not be identified as low quality using the above criterion, information across volatiles to determine if one of the three technical replicate runs consistently yielded low quality data points across volatiles was used. In this case, the technical replicate corresponding to the low quality run was excluded for volatiles with CV > 50% for the date palm sample.

After cleaning of the raw SPME/GC mass spectroscopy (SPME/GCMS) data, the means of the technical replicate m_i_ values were calculated per volatile per sample. These values are estimates of the number of butanol equivalents of each volatile per fruit sample with units μg/100 g and were included as trait values in a volatile 148 × 80 phenotype matrix with 3.28% (412/12,580) missing data. This matrix was used in downstream analysis of volatiles. Finally, a second matrix was created for comparison of volatiles to other fruit traits collected for a subset of the same date palm samples. The volatile matrix was merged with fruit organic acid, sugar composition, and fruit color data from Hazzouri et al. (2019) and then subsetted to exclude clonal replicates (see below) and remove samples with volatile phenotypes but without whole genome resequencing data.

### Volatile Data Analysis

**FIGURE 1:**
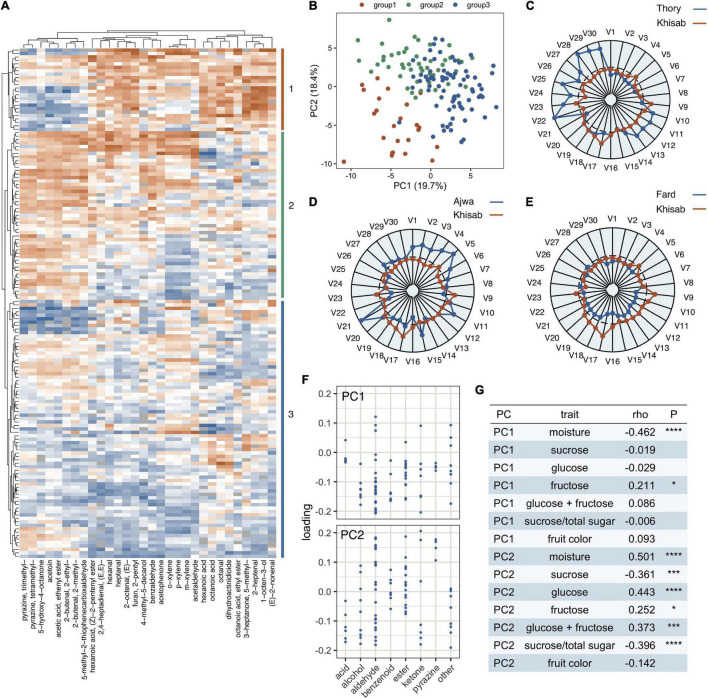
Variation in volatile composition of fruits from 148 date palms (*Phoenix dactylifera*). **(A)** Hierarchical clustering of date palm samples (vertical axis) and volatiles (horizontal axis) based on 30 volatiles. The heatmap represents transformed volatile concentrations (orange = high, blue = low). **(B)** PCA of 148 date palms based on 80 volatiles. **(C–E)** Radar plots contrasting volatile composition in “Thory,” “Ajwa,” and “Fard” varieties representing groups 1,2 and 3, respectively, contrasted with the “Khisab” variety. Volatiles (V1-30) correspond to the 30 volatiles in **(A)** read from left to right. **(F)** Loadings for axes 1 and 2 of the PCA in **(B)** for volatiles categorized by their class as defined in Table 1. **(G)** Table of Spearman’s Correlations of fruit traits and volatiles across date palm samples. Statistical significance is represented as *P < 0.05, ***P < 0.0005, and ****P < 0.00005, All analyses in **(A–F)** were conducted on log-transformed values scaled by Z-score transformation separately for each volatile. Outlier Z-score values were collapsed to –3 and 3 prior to clustering, heatmap, and radar plot production.

We ran principal component analysis (PCA) with the prcomp function in R (version 4.1.1). The input matrix to the PCA was modified by (i) imputing missing data with the median value calculated separately for each volatile, (ii) adding a constant (i.e., the minimum non-zero value) to all observations for any volatile with one or more zero values, (iii) taking the logarithm of the value, and (iv) both centering and scaling the values in the call to the prcomp function. PC1 (19.3%) and PC2 (18.7%) explained similar percentages of the overall variation. Thirty volatiles for hierarchical clustering in Figure 1A were chosen, by identifying the 15 volatiles with most extreme positive or negative loadings (i.e., absolute value) on each axis.

**TABLE 1:** Date palm fruit volatiles identified by SPME/GC-MS and included in the present study.

Volatile	Formula	RI Exp	Ri Lit^a^	CAS registry no.	Volatile studies^b,c,d^
**Acids**					
*Acetic acid	C_2_H_4_O_2_	1,453	1,449	64-19-7	
Decanoic acid	C_10_H_20_O_2_	2,281	2,276	334-48-5	
Hexanoic acid	C_6_H_12_O_2_	1,876	1,866^e^	142-62-1	E
Hexanoic acid, 2-ethyl-	C_8_H_16_O_2_	1,973	1,960	149-57-5	C
Nonanoic acid	C_9_H_18_O_2_	2,166	2,171	112-05-0	
Octanoic acid	C_8_H_16_O_2_	2,063	2,060	124-07-2	C
**Alcohols**					
Benzyl alcohol	C_7_H_8_O	1,899	1,890^e^	100-51-6	G
Ethanol	C_2_H_6_O	985	932	64-17-5	G,J
Hexanol	C_6_H_14_O	1,366	1,355	111-27-3	A,B,C,D,I,J
4-methyl-5-decanol	C_11_H_24_O	1,656	–	213547-15-0	
(E,E)-3,5-octadien-2-ol	C_8_H_14_O	1,405	–	69668-82-2	
Octanol	C_8_H_18_O	1,577	1,557	111-87-5	A,B,C,D,E,H,I,J
1-octen-3-ol	C_8_H_16_O	1,455	1,450	3391-86-4	A,B,C,D,E,I
2-octen-1-ol, (E)-	C_8_H_16_O	1,644	1,626^e^	18409-17-1	D
Phenylethyl alcohol	C_8_H_10_O	1,930	1,906	60-12-8	A,C,D,E,I
**Aldehydes**					
Acetaldehyde	C_2_H_4_O	720	702	75-07-0	G
*Benzaldehyde	C_7_H_6_O	1,511	1,520	100-52-7	A,C
Butanal, 2-methyl-	C_5_H_10_O	916	914	96-17-3	E,J
Butanal, 3-methyl-	C_5_H_10_O	919	918	590-86-3	
2-Butenal, (E)-	C_4_H_6_O	1,030	1,039	123-73-9	J
2-Butenal, 2-ethenyl-	C_6_H_8_O	1,270	1,303	20521-42-0	
2-Butenal, 2-methyl-	C_5_H_8_O	1,077	1,095	1115-11-3	
2-Butenal, 2-ethyl-	C_6_H_10_O	1,143	1,145	19780-25-7	
β-cyclocitral	C_10_H_16_O	1,619	1,611	432-25-7	A,C,D,E,I
*2-Furancarboxaldehyde, 5-methyl-	C_6_H_6_O_2_	1,569	1,570	620-02-0	G
*Furan-3-carboxaldehyde	C_5_H_4_O_2_	1,463	1,454	498-60-2	
1H-pyrrole-2-carboxaldehyde, 1-ethyl-	C_7_H_9_NO	1,613	1,610	2167-14-8	
2,4-Heptadienal, (E,E)-	C_7_H_10_O	1,490	1,495	4313-03-5	B,C
*Heptanal	C_7_H_14_O	1,178	1,184	111-71-7	B,C,E,H,J
2-Heptenal	C_7_H_12_O	1,323	1,323	2463-63-0	B
Hexanal	C_6_H_12_O	1,084	1,083	66-25-1	A,B,C,D,H,I,J
(E)-2-Hexenal	C_6_H_10_O	1,216	1,216	6728-26-3	B,C
5-Methyl-2-thiophene carboxaldehyde	C_6_H_6_OS	1,717	1,735^e^	13679-70-4	
*Nonanal	C_9_H_18_O	1,393	1,391	124-19-6	A,B,D,H,I,J
(E)-2-Nonenal	C_9_H_16_O	1,506	1,537	18829-56-6	B,C,D,E,G
Octanal	C_8_H_16_O	1,295	1,289	124-13-0	A,B,C,D,G,H,J
2-Octenal, (E)-	C_8_H_14_O	1,426	1,429	2548-87-0	B,C,D,I
2-Phenyl-2-butenal	C_10_H_10_O	1,937	1,929	4411-89-6	
**Esters**					
Acetic acid, ethenyl ester	C_4_H_6_O_2_	902	890	108-05-4	
Acetic acid, butyl ester	C_6_H_12_O_2_	1,072	1,074	123-86-4	G
Butanoic acid, butyl ester	C_8_H_16_O_2_	1,219	1,220	109-21-7	
Decanoic acid, methyl ester	C_11_H_22_O_2_	1,613	1,593	110-42-9	C,D
Dodecanoic acid, methyl ester	C_13_H_26_O_2_	1,836	1,834^e^	111-82-0	D
Ethyl acetate	C_4_H_8_O_2_	902	888	141-78-6	A,D,E,G,I,J
*Hexadecanoic acid, methyl ester	C_17_H_34_O_2_	2,212	2,208	112-39-0	
Hexadecanoic acid, ethyl ester	C_18_H_36_O_2_	2,255	2,251	628-97-7	E,I
Hexanoic acid, (Z)-2-pentenyl ester	C_11_H_20_O_2_	1,668	–	74298-89-8	
Nonanoic acid, methyl ester	C_10_H_20_O_2_	1,499	1,491	1731-84-6	C
Octadecanoic acid, ethyl ester	C_20_H_40_O_2_	2,451	2,451	111-61-5	G
*Octadecanoic acid, methyl ester	C_19_H_38_O_2_	2,419	2,418	112-61-8	G
8E,11E-Octadecadienoic acid, methyl ester	C_19_H_34_O_2_	2,477	–	56599-58-7	F
Octanoic acid, ethyl ester	C_10_H_20_O_2_	1,439	1,435	106-32-1	C,D,E,I
Pentadecanoic acid, methyl ester	C_16_H_32_O_2_	2,110	2,108	7132-64-1	
Tetradecanoic acid, methyl ester	C_15_H_30_O_2_	2,024	2,005	124-10-7	D
**Ketones**					
*Acetoin	C_4_H_8_O_2_	1,290	1,284	513-86-0	
Acetophenone	C_8_H_8_O	1,650	1,647	98-86-2	C,G
Ethanone, 1-(1H-pyrrol-2-yl)	C_6_H_7_NO	1,985	1,973	1072-83-9	
3-Heptanone, 5-methyl-	C_8_H_16_O	1,256	1,265	541-85-5	
5-Hepten-2-one, 6-methyl-	C_8_H_14_O	1,340	1,338	110-93-0	A,B,C,D,I,J
5-Hydroxy-4-octanone	C_8_H_16_O_2_	1,423	1,443	496-77-5	
β-ionone	C_13_H_20_O	1,947	1,971	79-77-6	A,B,C,D,E,I,J
**Pyrazines**					
Pyrazine, 3,5,-diethyl-2-methyl-	C_9_H_14_N_2_	1,516	1,496	18138-05-1	
Pyrazine, 2-ethyl-6-methyl-	C_7_H_10_N_2_	1,386	1,386	13925-03-6	
Pyrazine, tetramethyl-	C_8_H_12_N_2_	1,477	1,469	1124-11-4	
*Pyrazine, trimethyl-	C_7_H_10_N_2_	1,405	1,402	14667-55-1	
**Benzenoids**					
Ethylbenzene	C_8_H_10_	1,130	1,129	100-41-4	
p-cymene	C_1_0H_14_	1,261	1,272	99-87-6	J
Styrene	C_8_H_8_	1,254	1,261	100-42-5	D,F
Toluene	C_7_H_8_	1,030	1,042	108-88-3	
*m*-xylene	C_8_H_10_	1,139	1,143	108-38-3	
*o*-xylene	C_8_H_10_	1,171	1,186	95-47-6	
*p*-xylene	C_8_H_10_	1,121	1,138	106-42-3	F
**Other**					
2(4H)-benzofuranone, 5,6,7,7a-tetrahydro-4,4,7a- trimethyl-, (R)-	C_11_H_16_O_2_	2,290	2,325^e^	17092-92-1	C
γ-butyrolactone	C_4_H_6_O_2_	1,622	1,632	96-48-0	A
Furan, 2-pentyl-	C_9_H_14_O	1,229	1,231	3777-69-3	C,D
1H-pyrazole, 4,5-dihydro-5,5-dimethyl-4-isopropylidene-	C_8_H_14_N_2_	1,396	–	106251-09-6	
2-Methoxy-4-vinylphenol	C_9_H_10_O_2_	2,167	2,188	7786-61-0	
Phenol, 4-ethyl-2-methoxy	C_9_H_12_O_2_	2,167	2,188	2785-89-9	A,E,I
2-Pyrrolidinone	C_4_H_7_NO	2,029	2,017	616-45-5	
Vanillin	C_8_H_8_O_3_	2,531	2,540^e^	121-33-5	F

*Stereoisomers are indicated where they could be resolved. For compounds with multiple possible stereoisomers, reference to a compound in a prior study may or may not refer to the same stereoisomer detected in the present work.*

*Major volatile in the present study.

*^a^RI lit retention index coming from the NIST 14 database.*

*^b^Compound previously reported in date fruit by A = El Arem et al. (2011), B = Reynes et al. (1996), C = Khalil et al. (2017), D = Mezroua et al. (2017), E = Hatem et al. (2018), F = Arif and Lombarkia (2018), G = Narain (2007), H = Jaddou et al. (1984), I = El Arem et al. (2012), J = Harrak et al. (2005).*

*^c^No overlapping compounds were found with Siddeeg et al. (2019) and Aldulaimi et al. (2020).*

*^d^Acetic acid has previously been reported in non-volatile studies and is not considered novel to this study.*

*^e^RI lit retention index coming from Pubchem.*

Heatmaps and associated hierarchical clustering was performed using the ComplexHeatmap (v. 2.8.0; Gu, 2016) package in R. Prior to clustering and heatmap generation, volatile trait values were adjusted by adding a constant to traits with zero values (using the same procedure as in the PCA) followed by Z-score transformation of log-transformed values. Z-score values less than –3 or greater than 3 were set to –3 and 3, respectively prior to calling the Heatmap function. Hierarchical clustering on both the sample and volatile axis of the heatmap was performed using Euclidean distances.

Clustering of volatiles was performed with the pvclust package (v. 2.2-0) in R using 1—Spearman’s rank correlation as the distance measure. Node support was estimated as approximately unbiased (“AU”; see pvclust documentation) *P*-values obtained from 1,000 bootstrap replicates. The output was then plotted using the dendextend R package (v. 1.15.1) with pvclust_show_signif argument alpha = 0.05 to highlight nodes supported at this level. Manipulation of correlation matrices (e.g., Supplementary Table 2) was performed with the corrr R package (v. 0.4.3).

### SNP Processing

Unprocessed SNPs from Hazzouri et al. (2019) were filtered to remove variants mapping to the chloroplast genome followed by use of VariantFiltration and SelectVariants (GATK, v. 4.1.9.0) tools to retain only biallelic SNPs, exclude SNPs within 10 bp of indels in the unfiltered original call set, remove SNPs with a genotype call rate of less than 85%, and to exclude variants meeting the following conditions: QUAL < 30.0, DP < 785 (i.e., SNPs with less than 5X coverage averaged across samples), DP > 2063 (i.e., SNPs with greater than 1.5X the coverage summed across samples; The 1000 Genomes Project Consortium), QD < 5.0, SOR > 3.0, FS > 60.0, MQ < 40.0, MQRankSum < –12.5, ReadPosRankSum < –8.0. Variant Call Format (VCF) tag definitions can be found in the archived VCF file. SNPs not in Hardy-Weinberg equilibrium (*P* < 0.05) were subsequently removed using vcftools (v. 0.1.14). Imputation was performed on this call set with Beagle (v4.1; Browning and Browning, 2016) using the -gl option. This approach uses the genotype likelihoods in the filtered VCF to call genotypes and uses LD information to improve genotype calls while imputing missing genotypes in the original GATK callset. Imputation was performed with the default uniform recombination rate of 1 cM/Mb and without a reference panel or pedigree information. This VCF was used to identify clonal samples (see below).

### Kinship Analysis

Kinship coefficients were estimated on the VCF with ngsRelate (v. 2) using command line arguments “-T GT -c 1.” Given an expected kinship for clones of 0.5, we identified 16 independent pairs (*n* = 32 samples) with kinship ∼0.5 and one set of three clones (*n* = 3 samples) consisting of two named varieties “Khenezi” (*n* = 2) and “Mablasi” (*n* = 1) (Supplementary Table 3). Therefore, 35 of the 148 samples were verified by genome sequencing as being members of a clone pair (*n* = 2) or group (*n* = 3) (Supplementary Table 3). We inspected the *khalal* stage fruits of inferred clones bearing different variety names and confirmed the fruits have the same color and shape. We conclude these samples are clones, but they may be either cases where the same clone was assigned different variety names or cases in which one of the samples had been mis-labeled on one of the two farms. Three other pairs of samples shared a variety name (i.e., “Deglet Al Emam,” “Nebtet Seif,” and “Nagal”) but only one of the samples was sequenced preventing confirmation of clonal identity.

### Clonal Analysis of Volatile Composition

We tested the hypothesis that vegetative clone pairs are more similar in their volatile profiles than expected by chance using a randomization procedure. Each replicate of the procedure consisted of randomly choosing n pairs of samples from the 148 samples in our volatile dataset and assigning them as pseudo-clone pairs, where n corresponds to the observed number of clones (Supplementary Table 3). We then calculated the Euclidean distance between volatile profiles separately for each pair of pseudo-clones and determined the mean for the n pseudo-clone pairs. This procedure was repeated 10,000 times. The *P*-value was calculated as the proportion of the 10,000 replicates where the mean Euclidean distance between pseudo-clone pairs was less than the observed mean. Samples considered clones in this analysis included only those that could be verified based on whole genome sequencing. Analysis of broad sense heritability was conducted using the approach of Einspahr et al. (1963) and Zsuffa (1975) on clones in our analysis represented by two palms. Analysis of Variance (ANOVA) summary statistics for the calculations were obtained using the aov function in the base R (v. 4.1.1) stats package and data tidying performed with the Broom package (v. 0.7.10) in R.

## Results and Discussion

### Variation in Fruit Volatile Composition

We measured volatile composition of fruit from 148 date palms representing 135 varieties on two farms in the United Arab Emirates using headspace SPME/GC mass spectroscopy (Supplementary Table 1). The two farms (the Al Falassi farm in Abu Dhabi and the Al Hamria farm in Ras Al-Khaimah) are pre-existing farms that are unique in that they contain a large number of varieties planted together; as far as is known, these are two of the largest living collections of date palm varieties that exists. The two farms differ in various aspects of farm management and are located ∼100 km apart; nevertheless the environments are sufficiently similar that fruit samples from these different farms have been successfully used together in genetic mapping studies (Hazzouri et al., 2019). Given the structure of the populations it was not possible to obtain biological replicates per genotype/variety for volatile levels, so our sampling is similar to wild sampling and at this level any difference between sample cannot be attributed solely to variety. In several genotypes, however, there were replicate trees in the two different farms and we could estimate genetic effects (see below).

Tree-ripened fruits were harvested at the fully ripened stage (i.e., *tamar* stage, indicated by fruit abscission) from an individual tree; individual fruits were pooled and volatiles semi-quantified to allow relative comparison of varieties in subsequent analysis. A total of 80 volatiles were recorded across samples in our volatilome analysis, including acids (6), alcohols (9), aldehydes (23), esters (16), ketones (7), pyrazines (4), benzenoids (7), and other compounds (8) (Table 1). Thirty of the volatiles in Table 1, excluding potentially novel isoforms (i.e., *m*- and *o*-xylene), have not previously been reported in date palm fruit. These include dominant compounds in our analysis such as hexadecanoic acid, methyl ester, other fatty acid esters, branch chain aldehydes, furans, pyrazines, pyrrols, pyrrazoles, and 2-methoxy-4-vinylphenol, an attractant of the red palm weevil (*Rhynchophorus ferrugineus*), a major pest of the date palm crop (Table 1). Dominant compounds are indicated in Table 1. However, we note that the semi-quantification approach we adopted allows comparisons across samples for each volatile, but prevents a rigorous assessment of the relative abundance of different volatiles or volatile classes. Finally, one of the volatile compounds—acetic acid, ethenyl ester—was detected in our study but we could not rule out that it was a contaminant from storing the harvested date fruits in plastic bags.

The volatiles detected in our study include a number of compounds that either have not previously been reported or were rarely detected in date palm fruit. For example, pyrazines are produced by non-enzymatic processes during heating (Garcia et al., 2020) or as products of fermentation (Müller and Rappert, 2010). This class of compounds have been rarely observed in date palm fruit (El Arem et al., 2012); in contrast we find that trimethyl pyrazine is among the most dominant volatiles in our study. In other cases, we did not detect volatiles reported as dominant in prior studies (e.g., 2-propanol and isopentyl alcohol, El Arem et al., 2012; methyl propionoate, Hatem et al., 2018). Differences in assay sensitivity, experimental protocols, and the analytical procedure are possible contributing factors, but our approach of harvesting mature, tree-ripened fruits may also contribute to this observation. Our goal was to sample each variety at the same developmental stage and apply the same experimental protocol, while minimizing post-harvest handling, which contrasts with other study designs where volatiles have been profiled in dried fruits or after harvesting at an earlier stage.

Two volatiles in our analysis, 2-methoxy-4-vinylphenol and 1-octen-3-ol are of interest in the context of date palm pest management. The red palm weevil (RPW) is a severe threat to the crop in date-growing regions (El-Sabea et al., 2009) and volatile attractants are a primary means of limiting crop loss. RPW are attracted to fermenting plant material and traps are often baited both with RPW aggregation pheromones and date fruit baits, that act synergistically to lure the weevils (Soroker et al., 2015; Oehlschlager, 2016). 2-methoxy-4-vinylphenol is an aggregation pheromone produced by RPW (El-Sayed, 2021) that we also report in date fruits for the first time. This observation raises the possibility that 2-methoxy-4-vinylphenol is a natural coattractant of RPW that may contribute to the effectiveness of date fruit baits (Soroker et al., 2015). 2-methoxy-4-vinylphenol is also produced by coconut palm (*Cocus nucifera* L.) bark where it acts as a natural attractant for RPW (Gunawardena et al., 1998). Another interesting volatile compound in the context of RPW is a 1-octen-3-ol, which is weevil repellant that reduces feeding and oviposition activity (Guarino et al., 2015). Further inquiry into these compounds and the causes of variation among varieties is warranted.

### Clustering of Volatile and Other Fruit Traits

PCA on the 80 volatiles collected from 148 date palm samples was conducted, and hierarchical clustering performed using 30 volatiles chosen based on their contribution to separating samples in the PCA. Membership of samples in the three basal-most groups in the hierarchical clustering (illustrated on the vertical axis of a heatmap, Figure 1A and Supplementary Figure 1) were used to color samples in the original PCA (Figure 1B). Interestingly, we do not find any individual samples, or groups of samples, as being distinct from others.

The profiles of 30 volatiles contributing most to diversity across samples in the PCA are shown for date palm samples labeled “Thory,” “Ajwa,” and “Fard” in contrast to the widely grown “Khisab” in Figures 1C–E. While differences among samples are primarily quantitative, some patterns are apparent. For example, “Thory” represents samples labeled as group 1 of the hierarchical clustering analysis (Figure 1A) and located primarily in the lower left quadrant of the PCA plot (Figure 1B). “Thory” and similar samples have consistently greater abundance of alcohols, aldehydes and carboxylic acids primarily with 6–8 carbon chains compared with other samples (Figures 1A,C–E and Supplementary Figure 1).

We determined if individual volatiles or volatile classes (e.g., alcohols; Table 1) disproportionately contribute to quantitative variation in volatile composition among samples by extracting the loadings on the first and second PC axes for each compound. Figure 1F shows that loadings are low for all volatiles on a scale from –0.3 to 0.3. Therefore, no individual volatile or class appears to contribute disproportionately to differences among samples in volatile composition in tree-ripened fruit. This is in contrast to results in Muscadine grapes, for example, where 29 volatile compounds are responsible for significant differences between 5 varieties (Deng et al., 2021).

To gain additional insight into factors that contribute to fruit volatile diversity, we tested for significant correlations between PC1 and PC2 from the volatile PCA and traits, including fruit moisture content, sugar composition and the color of the fruit epicarp (i.e., measured at the *khalal* stage Hazzouri et al., 2019). Fruit moisture content was negatively correlated with PC1 (*P* < 0.00005, Figure 1G) and positively with PC2 (P < 0.00005, Figure 1G). Glucose (*P* < 0.00005, Figure 1G) and glucose + fructose (*P* < 0.0005, Figure 1G) were positively correlated with PC2, while sucrose (*P* < 0.0005, Figure 1G) and sucrose/total sugar (*P* < 0.00005), Figure 1G) were negatively correlated with this PC. The opposing relationship of reducing and invert sugars with respect to PC2 are likely related to the inverse correlation between these sugars in date fruits (Hazzouri et al., 2019). Fruit color was not associated with either PC axis (Figure 1G).

The correlations between PC2 with both glucose and sucrose (Figure 1G) implies that sugar composition is associated with the mixture of volatiles in ripened date palm fruits. Volatile composition as measured by PC1 and PC2 is also associated with moisture in our data (Figure 1G), although its unclear how this measure relates to the organoleptic properties of dry, semi-dry, and soft textures often used to characterize dried date fruits. Nevertheless, these correlations imply that differences among tree-ripened fruits in moisture and sugar content may be associated with system-wide metabolic differences in both volatile and non-volatile compounds that extend beyond sugar metabolism and the cellular processes controlling fruit moisture. This could be explained by differences in moisture content where the metabolism of fruits with low moisture content is arrested at an earlier stage than fruits with higher moisture content, which experience a greater degree of metabolic turnover during ripening (Diboun et al., 2015). In addition to correlations between volatiles and non-volatile traits, interesting correlations are found between specific volatiles that may reflect shared or distinct metabolic synthetic/catabolic pathways (see below).

### Genetic Basis of Volatile Composition: Clonal Analysis

A key question is whether differences in volatile profiles among samples reflect genetic or non-genetic factors. We examined whether genetic factors contribute to volatile levels by capitalizing on clones in our analysis which originate from the practice of vegetatitve propagation in date palms (Chao and Krueger, 2007). We analyzed whole genome sequence data from Hazzouri et al. (2019) to confirm the clonal status of elite commercial cultivars “Fard,” “Gharra,” “Khalas,” “Barhi,” “Khisab,” “Khenezi,” “Sultana,” “Chichi,” and “Abou Kibal” each of which is represented by one palm on each of the two farms in our sampling populations. These samples had kinship coefficients estimated from whole genome sequences of ∼0.5 confirming their clonal identity (Supplementary Table 3). In addition to these clones, kinship analysis also revealed eight additional pairs of clones that bear different variety names (e.g., “Jeish Fatima” and “Jeish Mohammad Khalaf”), but had kinship coefficients of ∼0.5.

To test for a genetic effect on volatile composition, we asked if pairs of clones collectively have more similar volatile profiles then expected by chance. Using a randomization procedure, we found that the mean Euclidean distance between volatile profiles of pairs of clones bearing the same name (*n* = 10) were significantly less than expected by chance (*P* < 0.01). Application of this same procedure to all clones (*n* = 16 pairs), including those bearing different variety names, indicated these samples also have more similar volatile profiles between pairs of clones than expected by chance (*P* < 0.05). Thus, the shorter than expected mean pairwise volatile profile distance of clonal samples supports a genetic effect on volatile composition.

**FIGURE 2:**
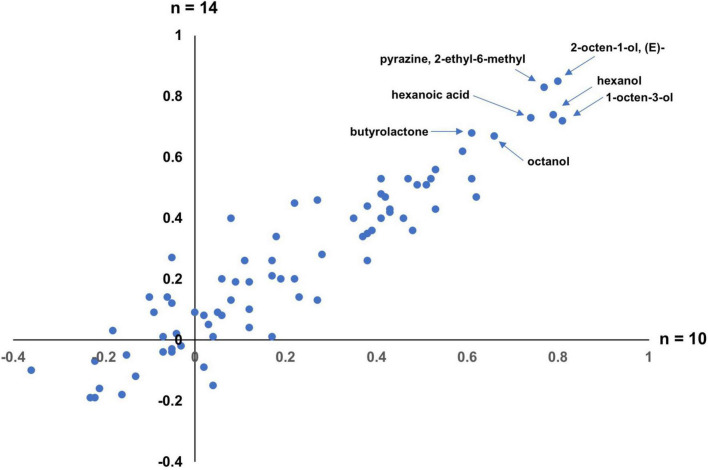
Estimates of broad-sense heritabilities (*H*^2^) for volatiles. The *x*-axis are the estimates for the *n* = 10 clonal pair set and the *y*-axis for the *n* = 14 clonal pair set. Volatiles with high *H*^2^ estimates are highlighted. The three volatiles whose estimates do not correlate well between clonal sets (2-pyrrolidinone, benzyl alcohol, 2,4-heptadienal, (E,E)−) are not included.

We took advantage of clonal pairs to estimate broad-sense heritabilities, *H*^2^, of each volatile (Einspahr et al., 1963; Zsuffa, 1975). We estimated *H*^2^ separately for the two overlapping sets of clones. One set consisted of clonal pairs in which clones had the same variety name (*n* = 10 pairs) and the other included four additional clones where members of each clone had different names (*n* = 14 pairs) (Supplementary Table 4). We find that the *H*^2^ estimates using these two sets correlate well with each other (Figure 2), except for 3 volatiles (2-pyrrolidinone, benzyl alcohol, 2,4-heptadienal, (E,E)−) which we do not consider further. In the larger (*n* = 14) set, we find that 16 of the volatiles had negative heritability estimates (Supplementary Table 4), including m-,o- and p-xylene, and decanoic acid; these negative values may arise from the estimation procedure, but there is also some debate about the meaning of negative heritabilities (Steinsaltz et al., 2020), and we do not consider these estimates further. Of the remaining 61 volatiles with positive *H*^2^ values, they range from 0.01 to 0.85. It should be noted that the *H*^2^ values we estimate are probably less meaningful than the relative ranking of volatile heritabilities. In this regard, we note that volatiles with high estimated heritabilities (*H*^2^ > 0.6), including 2-octen-1-ol, (E)-, hexanoic acid and butyrolactone, consist of volatiles representing different classes of compounds (e.g., alcohols, acids, etc.) and there is no class of volatiles with consistently high or low estimated heritability.

### Correlations Among Volatiles

**FIGURE 3:**
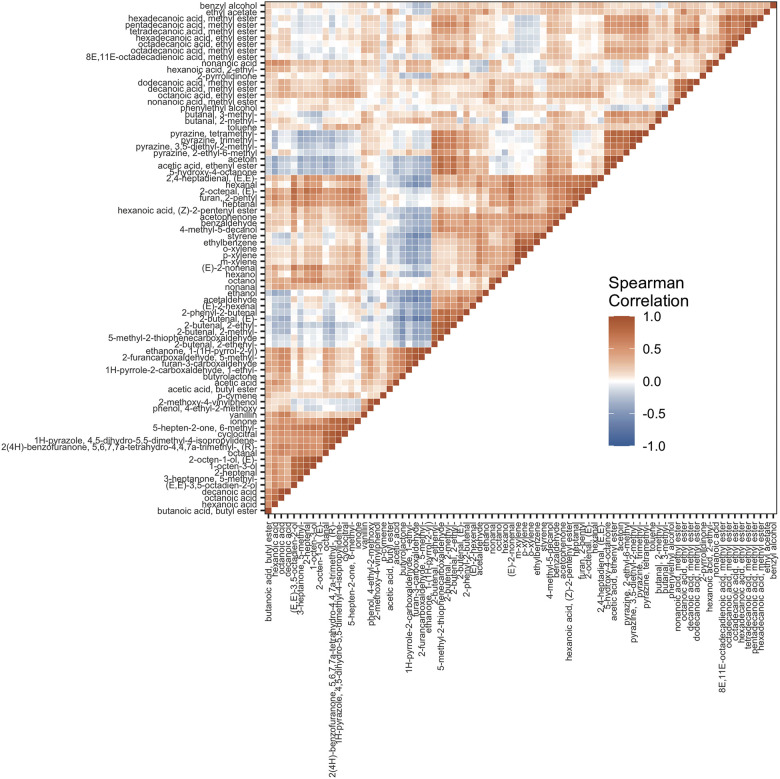
Heatmap of Spearman’s Rank Correlations between volatiles from 148 date palm samples. The order of volatiles from bottom to top in the vertical axis is repeated from left to right in the horizontal axis.

Correlations in metabolomic data can provide insight into metabolic networks (Camacho et al., 2005; Müller-Linow et al., 2007) and the biochemical pathways that produce poorly characterized volatile compounds (Tikunov et al., 2005; Mathieu et al., 2009). To gain insight into the date palm fruit volatiles, we follow the approach of Tikunov et al. (2005) and use our large sample of genotypes (i.e., 148 samples) to construct a matrix of Spearman’s rank correlations for the 80 volatiles (3,160 pairwise comparisons) (Figure 3 and Supplementary Table 2). This yielded 759 significant correlations (24%) after Bonferroni correction at α = 0.05 of which 681 were positive and 78 were negative.

**FIGURE 4:**
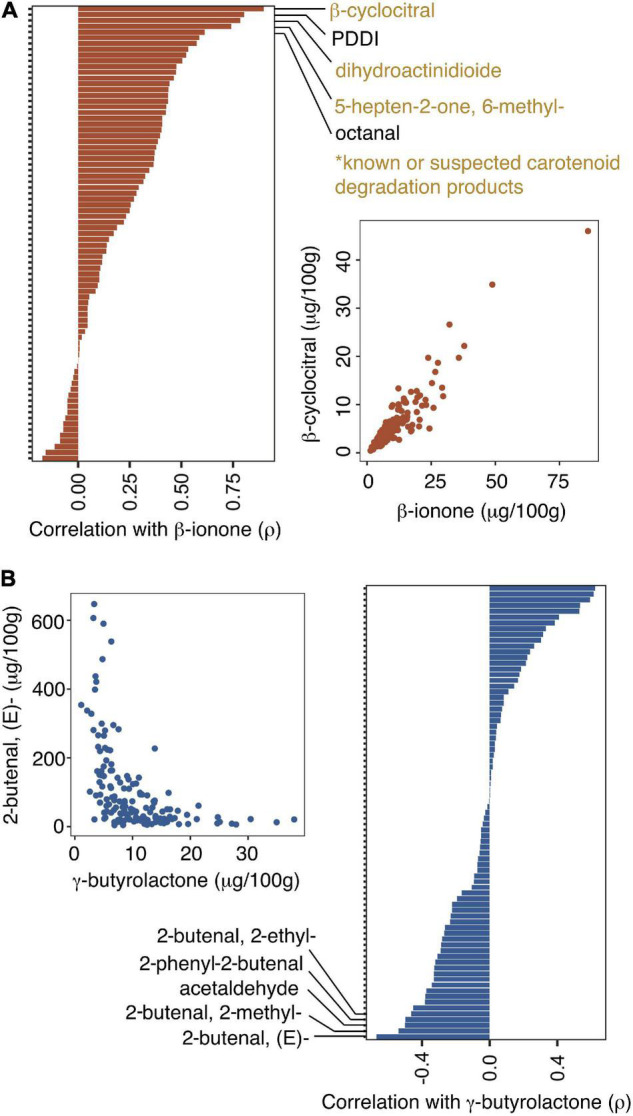
Spearman’s Rank Correlations among fruit volatile traits in 148 date palm fruit samples. **(A)** Correlations between β-ionone and other volatiles. Selected volatiles mentioned in the text are labeled. **(B)** Correlations between γ-butyrolactone and other volatiles. Selected volatiles mentioned in the text are labeled.

Positive correlations among metabolites suggest shared mechanisms of volatile production and clues to the pathway origins of compounds. In some cases, positive associations reflect shared pathways, while in others they reflect common regulation/genetic control. Many of the associations in our analysis reflect products of the same biochemical pathway. For instance, carotenoids accumulate at high concentrations in date palm fruits at early stages of fruit development and then are progressively broken down during maturation until they reach low concentrations in *tamar* stage fruits (Gross et al., 1983; Steingass et al., 2020). Degradation of these compounds produces apocarotenoids (Felemban et al., 2019). In tree-ripened date fruit, β-ionone, a breakdown product of β-carotene, is positively correlated with other apocarotenoids β-cyclocitral, 6-methyl-5-hepten-2-one, and dihydroactinidioide [i.e., 2(4H)-benzofuranone, 5,6,7,7a-tetrahydro-4,4,7a- trimethyl-, (R)-] consistent with their sharing precursors in the carotenoid pathway (Figures 3,4A, and Supplementary Table 2).

Negative correlations are less frequent in the volatile correlation matrix (Figure 3 and Supplementary Table 2); for some of these examples it could be an indication of competition for precursor molecules during volatile biosynthesis or represent alternate metabolic states of different fruit samples. For example, γ-butyrolactone, an important flavor compound in coffee and almonds, is negatively correlated with 2-butenal, (E)- (ρ = –0.67; *P* < 3.3 ×10^–20^, Figure 4B) and three other branched aldehydes that are structurally similar to 2-butenal, (E)- (Figure 4B). Other relatively strong negative correlations are between 2-butenal, (E)- and furan-3-carboxaldehyde (ρ = –0.68, *P* = 0.00) and 2-butenal, (E)- and 2-furancarboxaldehyde, 5-methyl- (ρ = –0.66, *P* < 1.97 × 10^–18^) and between furan-3-carboxaldehyde and acetaldehyde (ρ = –0.65; *P* = 0.00), ethanol (ρ = –0.57; *P* = 0.00), and styrene (ρ = –0.60; 7.5 × 10^–16^).

**FIGURE 5:**
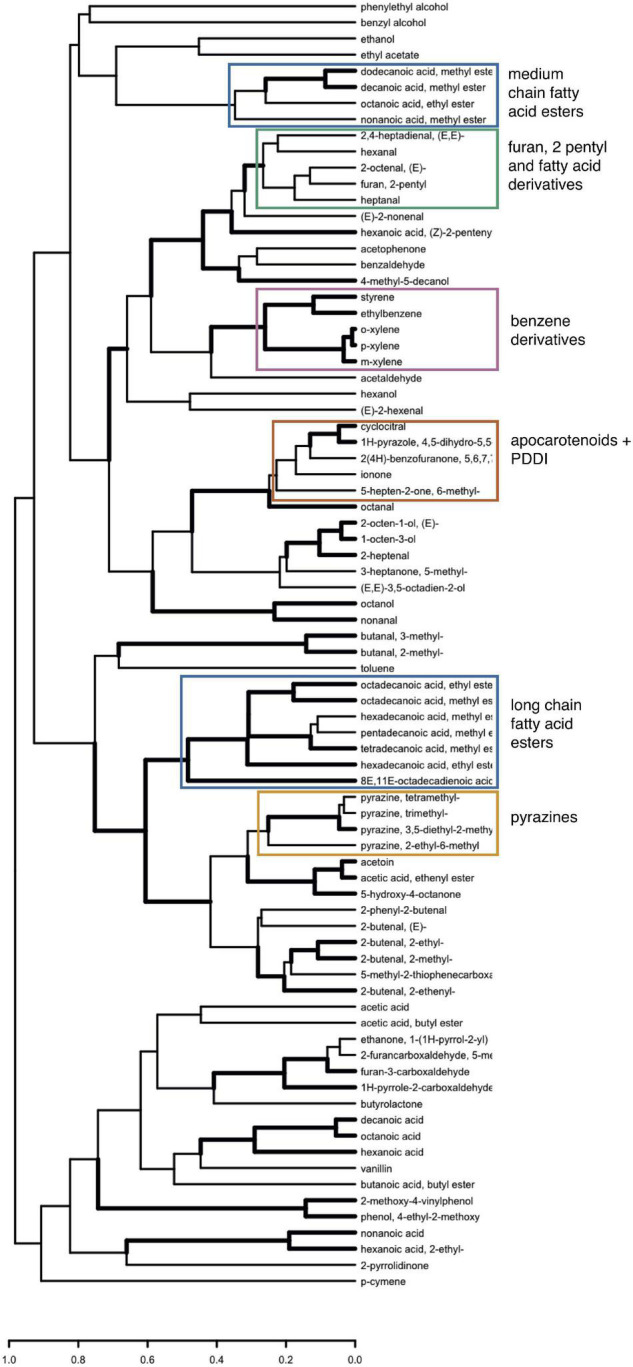
Hierarchical clustering analysis of 80 volatiles across date palm samples based on Spearman’s rank correlation. Distances used in clustering were calculated as 1—ρ. Edges represented by heavy lines are supported by approximately unbiased *P*-values < 0.05 inferred from bootstrapping.

Clustering analysis based on Spearman’s rank correlation distances (i.e., 1—ρ) provides an alternate means of gaining insight into volatile correlations (Tikunov et al., 2005; Mathieu et al., 2009). Groups of compounds that cluster together in this analysis suggest common regulation. For example, there is a large, well-supported, cluster of long chain fatty acid methyl or ethyl esters (Figure 5) with fatty acid chains ranging in length from 14 to 18 carbons. This cluster includes two of the dominant volatiles in our analysis, namely octadecanoic acid, methyl ester and hexadecanoic acid, methyl ester (the methyl esters of palmitic and stearic acids) (Table 1). By contrast, medium chain fatty acid esters with fatty acid chains ranging from 8 carbons (octanoic acid, ethyl ester) to 12 carbons (dodecanoic acid, methyl ester) form a cluster independent of the long chain fatty acid esters (Figure 5).

This analysis suggests that medium chain and long chain fatty acid ester volatiles are regulated independently. Since date palm fruit retain only trace amounts of triacylglycerides (TAGs) (Bourgis et al., 2011), a potential source of fatty acid precursors is the breakdown of membrane phospholipids during ripening. Fatty acids produced during fruit ripening may initially be liberated by lipase or phospholipase activity and then further metabolized via the lipoxygenase (LOX) or β-oxidation pathways to produce straight chain fatty acids, alcohols, and aldehydes. Differences in regulation of medium and long chain fatty acid esters could be related to differences in regulatory control of fatty acid precursors, differences in properties of enzymes such as alcohol acyl transferase (AAT) responsible for ester formation in fruits (Ueda and Ogata, 1977), or due to completely independent synthetic pathways of medium and long chain esters possibly involving microbes in ripening fruit. For example, medium chain fatty acid esters responsible for the fruity notes in wines are yeast secondary metabolites produced during fermentation (Hu et al., 2018). Support for distinct clusters of long chain and medium chain fatty acid esters suggests common regulation within each class, but independent regulation between classes.

Other clusters include pyrazines, benzene derivatives, and apocarotenoids (Figure 5). Apocarotenoids β-ionone, β-cyclocitral, 5-hepten-2-one, 6-methyl, and dihydroactinidioide cluster with 1H-pyrazole, 4,5-dihydro-5,5-dimethyl-4-isopropylidene- and octanal (Figure 5). While the clustering of apocarotenoids is consistent with a shared carotenoid degradation pathway, the cause for clustering with unrelated compounds including octanal is less clear, although similar observations have been reported elsewhere (Mathieu et al., 2009; Rambla et al., 2017). Finally, 2-pentylfuran is found in a well-supported cluster with fatty acid derivates supporting a connection between this compound and fatty acid degradation (Tikunov et al., 2005; Rambla et al., 2017).

### Correlations Among Volatiles, Organic Acids, Sugars, and Color Traits

Above, we reported correlations between sugar and moisture traits and PCs in the volatile PCA. Here, we test for correlations of these traits with individual volatiles, which have been reported previously for volatiles and organic acids and volatiles and fruit color (Ghnimi et al., 2018). We found no significant correlations between fruit color or anthocyanin content and the 80 volatiles after Bonferroni correction at α = 0.05 (Supplementary Table 2). We also tested for correlations between volatiles, sugars, and organic acids. We found a number of weak negative correlations between sucrose and some volatiles including 5-hydroxy-4-octanone (ρ = –0.53; *P* < 3.5 × 10^–9^). Correlations between all traits in our analysis are presented in Supplementary Table 2.

## Summary

Although volatile organic compounds are believed to underlie aroma and taste among varieties/cultivars of domesticated fruit tree species, there is remarkably few studies on fruit volatile diversity in these crops. Our study suggests substantial diversity in fruit volatile compounds between date palm varieties; such variation has also been observed in other fruit crop species such as Muscadine grapes (Deng et al., 2021), mangoes (Li et al., 2017), and strawberries (Zhao et al., 2020; Fan et al., 2021).

Date palm fruits differ in a number of commercially important traits, such as sugar content, which our analysis suggests is also correlated with volatile composition. Non-genetic factors, including subtle or cryptic differences in developmental stage at time of harvest and environmental effects, likely contribute to volatile trait variation. Such effects may account for the observation that some samples (e.g., “Abou Kibal”) have relatively large volatile profile distances between clonal pairs of samples (Supplementary Figure 1). Nevertheless, our clonal analysis indicates that fruit volatile diversity may indeed have a genetic basis, as date palm clones grown in different farms are more likely to share volatile profiles compared to non-related varieties. We have used this clonal structure to get estimates of broad-sense heritabilities for different volatile components, and we identified several volatiles with greater heritabilities compared to others within the sample sets. Identifying the genes underlying volatile composition is an obvious next step; unfortunately our sampling procedure as well as limited sample size precludes a genetic mapping analysis, and a full genetic study must await the availability of larger mapping populations.

The chemical basis of aroma has not been clearly defined in date palm, but appears to be explained by a complex mixture of volatiles. Presently, we know very little about which volatiles contribute to flavor perception. Hatem et al. (2018) suggested that alcohols and terpenes determine the herbaceous, fruity, citrus, floral and fungal aromas of date fruits and that 2-hexen-1-ol, 1-hexanol, and phenylethanol probably contribute to green, herbal, and floral aromas. El Arem et al. (2011) attributed the fresh and green notes of dates to nonanal and decanal straight chain aldehydes, of which the former was also detected in our study. In apples, approximately 350 volatiles have been reported (Song and Forney, 2008), but only around 20 are considered important for the distinctive flavors of apple varieties (Dixon and Hewett, 2000). We suspect this is similar in date palms and that only a small percentage of the volatiles we report impact flavor perception. Further advances in date fruit flavor research would benefit from sensory analysis that combines measurements of volatile, organic acid, and sugar composition in individual varieties with human sensory panels which is necessary to establish which compounds are most important in flavor perception (Ismail et al., 2001).

## Data Availability Statement

The original contributions presented in the study are publicly available. This data can be found here: 10.5061/dryad.mw6m905z8.

## Author Contributions

KH, ML, KA, and MP designed the research. KH and AL collected samples. ML performed headspace GC/MS and produced the raw volatile data. JF, ML, MP, SF, MG-B, and TC performed data collection and interpretation. JF performed the data analysis. JF and MP wrote the manuscript. All authors contributed to the article and approved the submitted version.

## Conflict of Interest

The authors declare that the research was conducted in the absence of any commercial or financial relationships that could be construed as a potential conflict of interest.

## Publisher’s Note

All claims expressed in this article are solely those of the authors and do not necessarily represent those of their affiliated organizations, or those of the publisher, the editors and the reviewers. Any product that may be evaluated in this article, or claim that may be made by its manufacturer, is not guaranteed or endorsed by the publisher.

## Footnotes

1
https://pubchem.ncbi.nlm.nih.gov/
