# Supplementary material for: Patterns of Volatile Diversity Yield Insights Into the Genetics and Biochemistry of the Date Palm Fruit Volatilome
Source: Front Plant Sci. 2022 Mar 14;13:853651. doi: 10.3389/fpls.2022.853651 (PMC8964304; doi:10.3389/fpls.2022.853651)

**SUPPLEMENTARY INFORMATION**

**Table S1.** **Date palm (*Phoenix dactylifera*) samples included in this study**.

| **Variety Name** | **ID^a^** | **Farm^b^** | **Volatiles^c^** |
| --- | --- | --- | --- |
| Al alik | Al_alik_rak | R | yes |
| Al waab | Al_waab | AF | yes |
| AZIZ | AZIZ_rak | R | yes |
| Deglet ALEMAM | Deglet_ALEMAM_rak | R | yes |
| Deglet Noor | Deglet_Noor_rak | R | yes |
| Hatimi | Hatimi_rak | R | yes |
| JABRI | JABRI_rak | R | yes |
| Jeish Habbash | Jeish_Habbash_rak | R | yes |
| Jeish Rabiei | Jeish_Rabiei | AF | yes |
| Jeish rashed | Jeish_rashed_rak | R | yes |
| Jeish stouh | Jeish_stouh_rak | R | yes |
| Nabtet Saif | Nabtet_Saif_rak | R | yes |
| Nagal | Nagal_rak | R | yes |
| Nebtet Shaheen | Nebtet_Shaheen | AF | yes |
| Ramah | PDAC253_Ram_S | AF | yes |
| Rij Al baneit | PDAC254_Rij_S | AF | yes |
| Sindy | PDAC255_Sin_S | AF | yes |
| Al maktoum | PDAC256_Alm_S | AF | yes |
| Hilali Saudi | PDAC257_Hil_S | AF | yes |
| Al Emam | PDAC258_Ale_S | AF | no |
| Zamili | PDAC260_Zam_S | AF | yes |
| Freija | PDAC261_Fre_S | AF | yes |
| Sheikha | PDAC262_She_S | AF | yes |
| Jeish Handal | PDAC263_Jei_S | AF | yes |
| Abou Labdehya | PDAC264_Lab_S | AF | yes |
| Mabrouma | PDAC265_Mab_S | AF | yes |
| Helwa | PDAC266_Hel_S | AF | yes |
| Rotana Al madina | PDAC267_Rot_S | AF | yes |
| Alhasawi | PDAC268_Has_S | AF | yes |
| Khoushana | PDAC270_Kho_S | AF | yes |
| Nbtet | PDAC271_Nbt_S | AF | yes |
| Tinana | PDAC272_Tin_S | AF | yes |
| Rariya | PDAC273_Rar_S | AF | yes |
| Fard Abyad | PDAC274_Far_S | AF | yes |
| Khadrawy | PDAC275_Kha_S | AF | yes |
| Nbetet Aldakyel | PDAC276_Ald_S | AF | yes |
| Khisyat Al mouznib | PDAC277_Khi_S | AF | yes |
| Um Ikbar | PDAC278_Umi_S | AF | yes |
| Nebtet seif | PDAC279_Nbt_S | AF | yes |
| Tanajib | PDAC280_Tin_S | AF | yes |
| Hijeri | PDAC281_Hje_S | AF | yes |
| Soukari Ahmar | PDAC282_Sou_S | AF | yes |
| Abou maan | PDAC283_Abo_S | AF | yes |
| Rotana | PDAC284_Rot_S | AF | yes |
| Soukari Iraqui | PDAC285_Sou_S | AF | yes |
| Soukary Saudi | PDAC286_Sou_S | AF | yes |
| Wanana | PDAC287_Wan_S | AF | yes |
| Safri | PDAC288_Saf_S | AF | yes |
| Ayasha | PDAC289_Aiy_S | AF | yes |
| Chichi | PDAC290_Chi_S | AF | yes |
| Nebtet Eid | PDAC291_Nbt_S | AF | yes |
| Medjhoolat Al jabal | PDAC292_Mej_S | AF | yes |
| Nebtet Rashed | PDAC293_Nbt_S | AF | yes |
| Ashala | PDAC294_Ash_S | AF | yes |
| Mordasinge | PDAC295_Mor_S | AF | yes |
| Nebtet zayed | PDAC296_Neb_S | AF | yes |
| Sbeika | PDAC297_Sab_S | AF | yes |
| Shagouni | PDAC298_Sag_S | AF | yes |
| Raziz | PDAC299_Raz_S | AF | yes |
| Maktoumi | PDAC300_Mak_S | AF | yes |
| Jeish Makran Al wan | PDAC301_Jei_S | AF | yes |
| Sultana | PDAC302_Sul_S | AF | yes |
| Sharaa | PDAC303_Sha_S | AF | yes |
| Shahl Alehseh | PDAC304_Sah_S | AF | yes |
| Mejhool | PDAC305_Med_S | AF | yes |
| Khlalife | PDAC306_Kha_S | AF | yes |
| Barhee | PDAC307_Bar_S | AF | yes |
| Anbara | PDAC308_Anb_S | AF | yes |
| Nebtet Abou hamad | PDAC309_Neb_S | AF | yes |
| Sagei | PDAC311_Sag_S | AF | yes |
| Lulu | PDAC312_Lul_S | AF | yes |
| Jeish ramli | PDAC313_jei_S | AF | yes |
| Madaain | PDAC314_Mad_S | AF | yes |
| Mansouriyya | PDAC315_Man_S | AF | yes |
| Nebtet Ali | PDAC316_Neb_S | AF | yes |
| Koroum Shahel | PDAC317_Kro_S | AF | yes |
| Khawaja | PDAC318_Kha_S | AF | yes |
| Shouweithi | PDAC319_Sho_S | AF | yes |
| Jeish Mohammad Kh | PDAC320_Jei_S | AF | yes |
| Zabad | PDAC321_Zab_S | AF | yes |
| Khisab | PDAC322_Khi_S | AF | yes |
| Nebtet Al mtawei | PDAC323_Nen_S | AF | yes |
| USA | PDAC324_Usa_S | AF | yes |
| Khenezi | PDAC325_Khe_S | AF | yes |
| Dahisiyya | PDAC326_Dah_S | AF | yes |
| Baydit falah | PDAC327_Bay_S | AF | yes |
| Miskaniyya | PDAC328_Mis_S | AF | yes |
| Khatiri | PDAC329_Kha_S | AF | yes |
| Khalas | PDAC330_Kha_S | AF | yes |
| Skebah | PDAC331_Ske_S | AF | yes |
| Azad | PDAC332_Aza_S | AF | yes |
| Fankhae | PDAC333_Fan_S | AF | yes |
| Rabiha | PDAC334_Rab_S | AF | yes |
| Ashawi | PDAC335_Ash_S | AF | no |
| Um Aldouhin | PDAC336_Umd_S | AF | yes |
| Dibbas | PDAC337_Dib_S | AF | yes |
| Ajwa | PDAC338_Ajw_S | AF | yes |
| Hbeisha | PDAC339_Hbe_S | AF | yes |
| Battash | PDAC340_Bat_S | AF | yes |
| Raysi | PDAC341_Ray_S | AF | yes |
| Gharra | PDAC342_Gha_S | AF | yes |
| Barni AI Ays | PDAC343_Bar_S | AF | yes |
| Shahouni | PDAC344_Sha_S | AF | no |
| Abou Jbeibil | PDAC345_Abo_S | AF | yes |
| Nebtet Kweity | PDAC346_Nbt_S | AF | yes |
| Nagal | PDAC347_Nag_S | AF | yes |
| Jeish Fatima | PDAC348_Jei_S | AF | yes |
| Salimiyat Aneyza | PDAC351_Sal_S | AF | yes |
| Loubana | PDAC352_Lou_S | AF | yes |
| Nimishi | PDAC353_Nim_S | AF | yes |
| houshana | PDAC354_Hou_S | AF | yes |
| Khashkar Hashkar | PDAC376_Kha_R | R | no |
| Saamaran | PDAC378_Saa_R | R | yes |
| Fard | PDAC379_Far_R | R | yes |
| Jeish Falka | PDAC380_Jei_R | R | yes |
| Um Aldouhin dhen | PDAC381_Uma_R | R | yes |
| Jeish sweihana Hamad | PDAC382_Jei_R | R | yes |
| Ibrahimi | PDAC383_Ibr_R | R | no |
| Breim | PDAC386_Bre_R | R | yes |
| Sultana | PDAC387_Sul_R | R | yes |
| Khad Al Faras | PDAC388_Kha_R | R | no |
| Zahidi | PDAC389_Zah_R | R | yes |
| Milkabi | PDAC390_Mal_R | R | yes |
| Halawi | PDAC391_Hal_R | R | yes |
| Khalas | PDAC394_Kha_R | R | yes |
| Khad AI Asad | PDAC395_Kha_R | R | yes |
| Jahl | PDAC396_Jah_R | R | yes |
| Hayani | PDAC397_Hay_R | R | no |
| Anwan | PDAC398_Anw_R | R | yes |
| Jeish Ali Abbas | PDAC399_Jei_R | R | yes |
| Ashhal | PDAC402_Ash_R | R | yes |
| Khadrawy | PDAC403_Kha_R | R | yes |
| Jeish samra | PDAC404_Jei_R | R | yes |
| Jeish Waeib | PDAC405_Jei_R | R | yes |
| Um Al salla | PDAC406_Jei_R | R | yes |
| Hilali Ahmar | PDAC407_Hil_R | R | yes |
| Zmouridi | PDAC410_Zou_R | R | yes |
| Dibbas | PDAC411_Dib_R | R | no |
| Barhee | PDAC412_Bar_R | R | yes |
| Thory | PDAC413_Tho_R | R | yes |
| Mablasi | PDAC414_Mab_R | R | yes |
| Msala | PDAC415_Msa_R | R | yes |
| Khisab | PDAC416_Khi_R | R | yes |
| Chichi | PDAC417_Chi_R | R | yes |
| Dayri | PDAC418_Day_R | R | yes |
| Jeish Mzamil | PDAC419_Jei_R | R | yes |
| Khatiri | PDAC422_Kha_R | R | yes |
| Abou Alouzouk | PDAC423_Abo_R | R | no |
| Ain Bakar | PDAC424_Ain_R | R | yes |
| Khenezi | PDAC425_Khe_R | R | yes |
| Maznani | PDAC426_Maz_R | R | no |
| Zagloul | PDAC427_Zag_R | R | yes |
| Jeish AI Saudiyya | PDAC431_Jei_R | R | yes |
| Abou Kibal | PDAC432_Abo_R | R | yes |
| Jeish Jaafar | PDAC433_Jei_R | R | yes |
| Gharra | PDAC434_Gha_R | R | yes |
| Skouti | Skouti_rak | R | yes |
| Soukar Masr | Soukar_Masr | AF | yes |

^a^Internal identifier

^b^AF= Ahmad Al Falassi Farm, Al Schweib, UAE, R = Al Hamria Farm, Ras-Al-Khaima, UAE

^c^Indicates if volatile data were collected

**Table S2. Trait correlation matrix. Spearman’s rank correlations are provided in the lower half matrix and raw P values upper.** Definitions of non-volatile traits in the table (e.g., RAB, MO, etc.) are defined in Hazzouri *et al*. (2019).

This table is included as a separate file.

**Table S3. Clonal pairs of samples identified in the date palm sampling panel identified from estimates of the kinship from whole genome re-sequencing data.**

| **Sample 1** | | | **Sample 2** | | |  |
| --- | --- | --- | --- | --- | --- | --- |
| **Variety** | **Identifier** | **Farm*** | **Variety** | **Identifier** | **Farm** | **Kinship** |
| Fard Abiad | PDAC274_Far_S | AF | Fard | PDAC379_Far_R | R | 0.508 |
| Gharra | PDAC434_Gha_R | R | Gharra | PDAC342_Gha_S | AF | 0.499 |
| Khatiri | PDAC329_Kha_S | AF | Khatiri | PDAC422_Kha_R | R | 0.498 |
| Khalas | PDAC330_Kha_S | AF | Khalas | PDAC394_Kha_R | R | 0.499 |
| Barhee | PDAC412_Bar_R | R | Barhee | PDAC307_Bar_S | AF | 0.500 |
| Khisab | PDAC416_Khi_R | R | Khisab | PDAC322_Khi_S | AF | 0.498 |
| Khenezi^b^ | PDAC425_Khe_R | R | Khenezi^b^ | PDAC325_Khe_S | AF | 0.500 |
| Sultana | PDAC387_Sul_R | R | Sultana | PDAC302_Sul_S | AF | 0.494 |
| Chichi | PDAC417_Chi_R | R | Chichi | PDAC290_Chi_S | AF | 0.499 |
| Sagae | PDAC311_Sag_S | AF | Sabbaka | PDAC297_Sab_S | AF | 0.497 |
| Zahidi | PDAC389_Zah_R | R | Loubana | PDAC352_Lou_S | AF | 0.499 |
| Amriki (USA) | PDAC324_Usa_S | AF | Medjool | PDAC305_Med_S | AF | 0.549 |
| Abou Kibal | PDAC432_Abo_R | R | Abou Kibal | PDAC345_Abo_S | AF | 0.500 |
| Mablasi^b^ | PDAC414_Mab_R | R | Khenezi^b^ | PDAC325_Khe_S | AF | 0.500 |
| Mablasi^b^ | PDAC414_Mab_R | R | Khenezi^b^ | PDAC425_Khe_R | R | 0.500 |
| Jeish Fatima | PDAC348_Jei_S | AF | Jeish Mohammad Khalaf | PDAC320_Jei_S | AF | 0.499 |
| Nebtet Masoudia | PDAC310_Neb_S^a^ | AF | Nbtet Rashed | PDAC293_Nbt_S | AF | 0.499 |
| Ashala | PDAC294_Ash_S | AF | Asiliya | PDAC259_Asi_S^a^ | AF | 0.500 |
| Hilali Ahmar | PDAC407_Hil_R | R | Shahouni | PDAC377_Sha_R^a^ | R | 0.499 |

^*^R = Al Hamria Farm, Ras-Al-Khaima, UAE; AF=Ahmad Al Falassi Farm, Al Schweib, UAE

^a^Volatile data not collected

^b^Three member clone

**Table S4. Broad-sense heritabilities (*H^2^*) based on analysis of 10 and 14 clonal pairs**

|  | **n = 10** | | | **n = 14** | | |
| --- | --- | --- | --- | --- | --- | --- |
| **Volatile** | **Clones df** | **Trees within clones df** | ***H^2^*** | **Clones df** | **Trees within clones df** | ***H^2^*** |
| (E,E)-3,5-octadien-2-ol | 9 | 9 | 0.49 | 13 | 14 | 0.47 |
| (E)-2-hexenal | 9 | 9 | -0.15 | 13 | 13 | -0.1 |
| (E)-2-nonenal | 9 | 7 | 0.41 | 13 | 10 | 0.4 |
| 1-octen-3-ol | 9 | 10 | 0.81 | 13 | 14 | 0.83 |
| 1H-pyrazole, 4,5-dihydro-5,5-dimethyl-4-isopropylidene- | 9 | 10 | 0.28 | 13 | 13 | 0.28 |
| 1H-pyrrole-2-carboxaldehyde, 1-ethyl- | 9 | 10 | 0.38 | 13 | 14 | 0.35 |
| 2-butenal, (E)- | 9 | 10 | 0.02 | 13 | 12 | 0.04 |
| 2-butenal, 2-ethenyl- | 9 | 9 | 0.08 | 13 | 14 | 0.12 |
| 2-butenal, 2-ethyl- | 9 | 10 | -0.04 | 13 | 14 | 0.01 |
| 2-butenal, 2-methyl- | 9 | 10 | -0.05 | 13 | 14 | -0.02 |
| 2-furancarboxaldehyde, 5-methyl- | 9 | 9 | -0.07 | 13 | 14 | -0.04 |
| 2-heptenal | 9 | 10 | 0.61 | 13 | 14 | 0.53 |
| 2-methoxy-4-vinylphenol | 9 | 10 | 0.51 | 13 | 14 | 0.47 |
| 2-octen-1-ol, (E)- | 9 | 7 | 0.8 | 13 | 14 | 0.74 |
| 2-octenal, (E)- | 9 | 10 | 0.46 | 13 | 14 | 0.44 |
| 2-phenyl-2-butenal | 9 | 10 | -0.05 | 13 | 14 | 0.01 |
| 2-pyrrolidinone | 8 | 4 | 0.99 | 13 | 10 | 0.85 |
| 2,4-heptadienal, (E,E)- | 9 | 6 | 0.7 | 13 | 14 | 0.67 |
| 2(4H)-benzofuranone, 5,6,7,7a-tetrahydro-4,4,7a-trimethyl-, (R)- | 9 | 10 | 0.53 | 13 | 13 | 0.51 |
| 3-heptanone, 5-methyl- | 9 | 9 | 0.53 | 13 | 14 | 0.51 |
| 4-methyl-5-decanol | 9 | 8 | -0.22 | 13 | 14 | -0.19 |
| 5-hepten-2-one, 6-methyl- | 9 | 10 | 0.23 | 13 | 13 | 0.26 |
| 5-hydroxy-4-octanone | 9 | 10 | 0.06 | 13 | 13 | 0.09 |
| 5-methyl-2-thiophenecarboxaldehyde | 9 | 10 | 0.42 | 13 | 14 | 0.42 |
| 8E,11E-octadecadienoic acid, methyl ester | 9 | 9 | 0.17 | 13 | 14 | 0.2 |
| acetaldehyde | 9 | 10 | 0.62 | 13 | 14 | 0.53 |
| acetic acid | 9 | 10 | 0.08 | 13 | 14 | 0.13 |
| acetic acid, butyl ester | 9 | 10 | -0.36 | 12 | 7 | -0.31 |
| acetic acid, ethenyl ester | 9 | 10 | 0.41 | 13 | 14 | 0.4 |
| acetoin | 9 | 10 | 0.12 | 13 | 14 | 0.14 |
| acetophenone | 9 | 10 | -0.1 | 13 | 12 | -0.07 |
| benzaldehyde | 9 | 10 | 0.43 | 13 | 14 | 0.43 |
| benzyl alcohol | 9 | 10 | 0.64 | 13 | 13 | 0.56 |
| butanal, 2-methyl- | 9 | 10 | -0.18 | 13 | 14 | -0.15 |
| butanal, 3-methyl- | 9 | 10 | 0.27 | 13 | 14 | 0.27 |
| butanoic acid, butyl ester | 9 | 10 | -0.05 | 13 | 14 | -0.03 |
| butyrolactone | 9 | 10 | 0.61 | 13 | 14 | 0.53 |
| cyclocitral | 9 | 10 | 0.37 | 13 | 14 | 0.34 |
| decanoic acid | 9 | 10 | -0.22 | 13 | 13 | -0.18 |
| decanoic acid, methyl ester | 9 | 10 | 0.04 | 13 | 14 | 0.08 |
| dodecanoic acid, methyl ester | 9 | 10 | -0.05 | 13 | 13 | 0.01 |
| ethanol | 9 | 10 | 0.22 | 13 | 10 | 0.26 |
| ethanone, 1-(1H-pyrrol-2-yl) | 9 | 10 | 0.03 | 13 | 14 | 0.08 |
| ethyl acetate | 9 | 10 | 0.41 | 13 | 13 | 0.4 |
| ethylbenzene | 9 | 10 | 0.17 | 12 | 13 | 0.19 |
| furan-3-carboxaldehyde | 9 | 10 | 0.06 | 13 | 14 | 0.1 |
| furan, 2-pentyl | 9 | 10 | -0.03 | 12 | 13 | 0.02 |
| heptanal | 9 | 10 | 0.48 | 13 | 14 | 0.46 |
| hexadecanoic acid, ethyl ester | 9 | 10 | 0.17 | 13 | 14 | 0.2 |
| hexadecanoic acid, methyl ester | 9 | 10 | -0.09 | 13 | 10 | -0.06 |
| hexanal | 9 | 10 | 0.43 | 13 | 14 | 0.43 |
| hexanoic acid | 9 | 7 | 0.74 | 13 | 14 | 0.68 |
| hexanoic acid, (Z)-2-pentenyl ester | 9 | 8 | 0.12 | 13 | 13 | 0.19 |
| hexanoic acid, 2-ethyl- | 9 | 9 | -0.16 | 13 | 14 | -0.12 |
| hexanol | 9 | 10 | 0.79 | 13 | 10 | 0.73 |
| ionone | 9 | 10 | 0.18 | 13 | 14 | 0.2 |
| m-xylene | 9 | 10 | -0.13 | 13 | 13 | -0.09 |
| nonanal | 9 | 10 | 0.47 | 13 | 14 | 0.45 |
| nonanoic acid | 9 | 7 | 0.38 | 13 | 14 | 0.36 |
| nonanoic acid, methyl ester | 9 | 10 | 0.27 | 13 | 14 | 0.26 |
| o-xylene | 9 | 10 | -0.21 | 13 | 14 | -0.16 |
| octadecanoic acid, ethyl ester | 9 | 9 | 0.09 | 13 | 12 | 0.13 |
| octadecanoic acid, methyl ester | 9 | 10 | 0.39 | 13 | 14 | 0.4 |
| octanal | 9 | 10 | 0.35 | 13 | 14 | 0.34 |
| octanoic acid | 9 | 9 | 0.05 | 13 | 13 | 0.09 |
| octanoic acid, ethyl ester | 9 | 10 | 0.12 | 13 | 14 | 0.14 |
| octanol | 9 | 10 | 0.66 | 13 | 14 | 0.62 |
| p-cymene | 9 | 9 | 0.02 | 13 | 14 | 0.05 |
| p-xylene | 9 | 10 | -0.23 | 13 | 13 | -0.19 |
| pentadecanoic acid, methyl ester | 9 | 10 | 0.22 | 13 | 14 | 0.26 |
| phenol, 4-ethyl-2-methoxy | 9 | 10 | 0.59 | 13 | 14 | 0.53 |
| phenylethyl alcohol | 9 | 10 | 0.52 | 13 | 14 | 0.48 |
| pyrazine, 2-ethyl-6-methyl | 9 | 10 | 0.77 | 13 | 14 | 0.72 |
| pyrazine, 3,5-diethyl-2-methyl- | 9 | 10 | 0 | 13 | 14 | 0.03 |
| pyrazine, tetramethyl- | 9 | 10 | -0.07 | 13 | 13 | -0.05 |
| pyrazine, trimethyl- | 9 | 10 | 0.11 | 13 | 13 | 0.14 |
| styrene | 9 | 10 | 0.04 | 13 | 14 | 0.09 |
| tetradecanoic acid, methyl ester | 9 | 10 | -0.06 | 13 | 13 | -0.04 |
| toluene | 9 | 10 | 0.19 | 13 | 14 | 0.21 |
| vanillin | 9 | 10 | 0.38 | 13 | 14 | 0.36 |

df = degrees of freedom


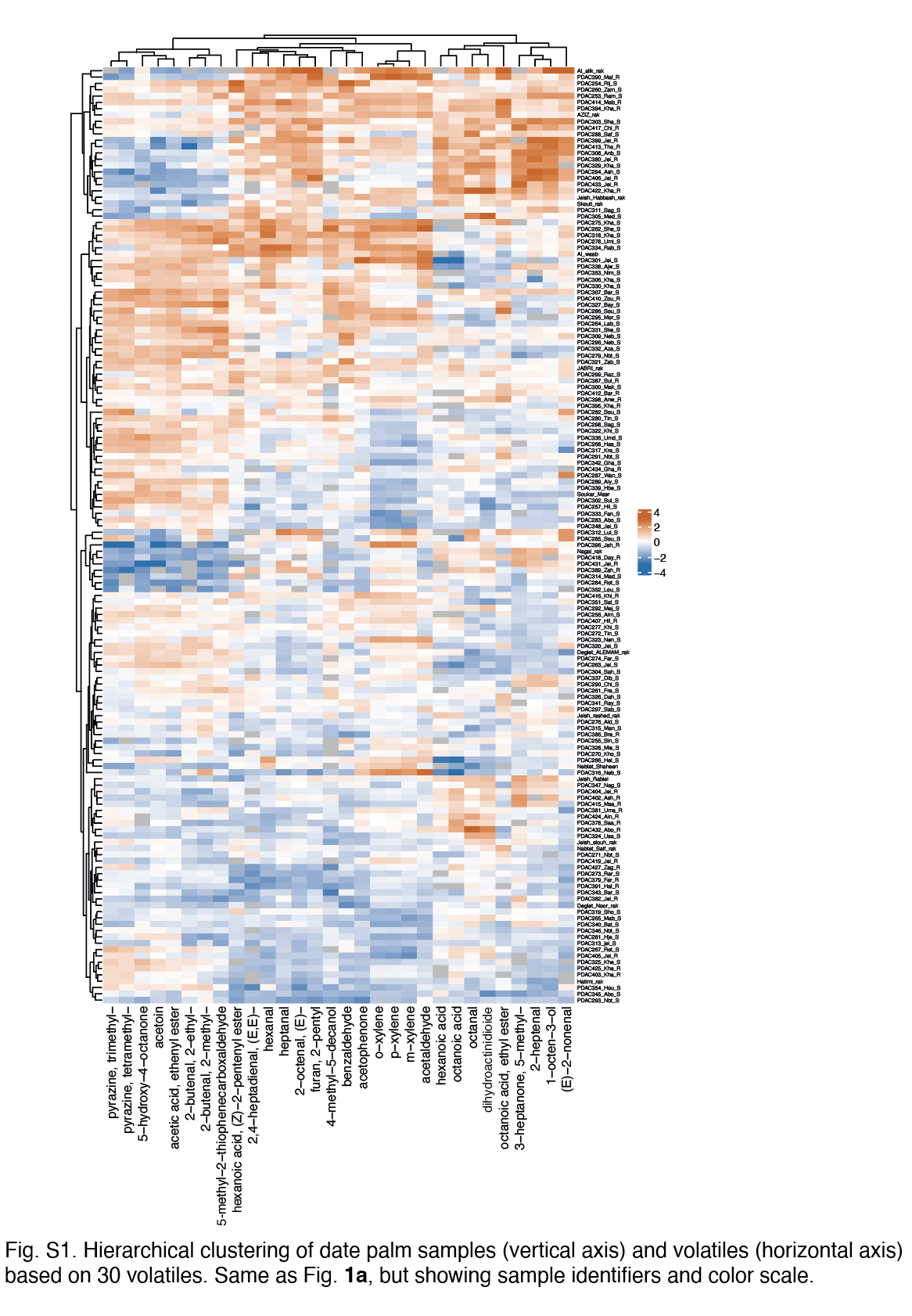

Supplement: Supplementary file 2 [file Data_Sheet_2.docx]
